# EHMTI-0361. Lack of differences in microrna expression profiles of blood cells in migraine

**DOI:** 10.1186/1129-2377-15-S1-H3

**Published:** 2014-09-18

**Authors:** M Vila-Pueyo, J Fernández-Morales, M Torres-Ferrus, J Álvarez-Sabin, P Pozo-Rosich

**Affiliations:** 1 Headache and Neurological Pain Group, Vall Hebron Research Institute, Barcelona, Spain; 2 Neurology, Vall Hebron Hospital, Barcelona, Spain

## Introduction

Migraine is a complex and very common neurological disorder. Several genome-wide significant loci have been described in migraine without explaining its whole genetic complexity. microRNAs are small non-coding RNAs involved in the regulation of gene expression that have become prominent candidates to explain the development of different neurological diseases and have also been described as key regulators in nociception.

## Aim

To detect microRNA expression differences involved in the susceptibility and chronification of migraine.

## Methods

20 migraineurs (5 episodic without aura, 5 chronic without aura, 5 episodic with aura, 5 chronic with aura) and 5 headache-free controls diagnosed by neurologists were included. In order to minimize differences, all 5 groups were homogenous regarding age (average 40.4 ±1.98), sex (3 women, 2 men), and a family history of migraine (first-degree relatives). Patients diagnosed with chronic migraine suffered from more than 15 days/month and patients diagnosed with episodic migraine had 1-2 days/month. Patients with aura suffered from more than 50% of attacks with aura.

RNA was extracted from peripheral blood mononuclear cells (PBMCs), microRNA expression profiles were determined and all possible comparisons were performed.

## Results

There were no statistically significant differences in any of the comparisons performed.

## Conclusions

This is the first microRNA analysis performed in migraine. The negative results indicate the lack of differences in microRNA expression in PBMCs in migraine. However, gene expression regulatory mechanisms such as microRNAs are tissue and cell specific, suggesting that differences in microRNA expression linked to migraine should be explored in brain tissue.

No conflict of interest.

